# Formulation and evaluation of ocean dynamics problems as optimization problems for quantum annealing machines

**DOI:** 10.1371/journal.pone.0326303

**Published:** 2025-06-26

**Authors:** Takuro Matsuta, Ryo Furue

**Affiliations:** 1 Faculty of Environmental Earth Science, Hokkaido University, Hokkaido, Japan; 2 JAMSTEC, Yokohama, Japan; University of Southern California, UNITED STATES OF AMERICA

## Abstract

Recent advancements in quantum computing suggest the potential to revolutionize computational algorithms across various scientific domains including oceanography and atmospheric science. The field is still relatively young and quantum computation is so different from classical computation that suitable frameworks to represent oceanic and atmospheric dynamics are yet to be explored. Quantum annealing (QA), one of the major paradigms, focuses on combinatorial optimization tasks. Given its potential to excel in NP-hard problems, QA may significantly accelerate the calculation of ocean and atmospheric systems described by the Navier-Stokes equations in the future. In this paper, we apply both QA and simulated annealing (SA), its classical counterpart, to a simplified ocean model known as the Stommel problem. We use the Stommel problem, which is not an NP problem and therefore does not benefit from QA today, just as an example, a first step in exploring QA for more intricate problems governed by the Navier-Stokes equations. We cast the linear partial differential equation governing the Stommel model into an optimization problem by the least-squares method and discretize the cost function in two ways: finite difference and truncated basis expansion. In either case, SA successfully reproduces the expected solution when appropriate parameters are chosen. In contrast, QA using the D-Wave quantum annealing machine fails to obtain good solutions for some cases owing to hardware limitations; in particular, the highly limited connectivity graph of the machine limits the size of the solvable problems, at least under currently available algorithms. Either expanding the machine’s connectivity graph or improving the graph-embedding algorithms would probably be necessary for quantum annealing machines to be usable for oceanic and atmospheric dynamics problems.

## 1. Introduction

The ocean and atmospheric circulations play a crucial role in shaping the climate system, necessitating accurate simulations of geophysical fluid dynamics for climate prediction. High-performance computing has successfully reproduced and predicted large-scale oceanic and atmospheric motions and climate changes [1–4]. However, scale separation does not exist for oceanic and atmospheric circulation and subgrid-scale parameterizations are not adequate for some problems. For example, the interaction between oceanic mesoscales, submesoscales, and internal waves, or interaction between coastal regions and the open ocean do not seem to allow for adequate subgrid-scale parameterizations. The only practical solution therefore seems to be to solve for all the relevant scales simultaneously. Until recently, Moore’s law had allowed us to increase grid resolution as computers became exponentially faster year by year. That is unfortunately no longer the case. The alternative is to rewrite our simulation programs for specific hardware to improve performance. Despite the human-resource cost to do so, the gain will not be systematic and will sooner or later diminish as the code gets more and more complicated [5].

Quantum computing may be regarded as this approach but for radically different hardware using radically different programming methods [6–9]. There are two leading paradigms of quantum computation: One is gate quantum computing and the other is quantum annealing (QA). The former constructs a series of quantum operations on a set of quantum bits or “qubits”, analogous to the logic circuit in classical computing [10]. The latter is designed to solve combinatorial optimization problems by controlling quantum ﬂuctuations [11,12]. As detailed below, in QA we “program” a physical system in such a way that it is a direct analogy of a mathematical problem.

While quantum gate–based systems remain far from practical, QA machines provided by D-Wave Inc. [13] have been put to practical use [14,15]. Notably, QA approaches have been tried in various industrial domains, including traffic flow optimization [16] and machine learning [17] (for a comprehensive review, refer to [15]). A potential advantage of QA is its potential speed in the future for large problems [6,18–20] although real annealing machines are currently not always efficient and often give solutions that include large errors due to noise on hardware. QA sometimes excels in NP problems but there is potential that it will be faster for other optimization problems in the future than classical computers [6,18]. In addition, QA machines possibly have higher power efficiency, i.e., computing power per watts, than classical supercomputers [21].

Prompted by these developments in quantum computing technology, we explore the potential of QA in solving problems in atmospheric and oceanic science. As far as we know, [22–24] are the only papers in that direction. Quantum computational techniques are unfamiliar to many atmospheric and ocean scientists and potential method to incorporate quantum techniques in model development is largely unexplored [9]. The aim of this paper is to bridge the gap between oceanography and quantum computational techniques by showing for the first time that a simple partial differential equation that models ocean circulation (see below) can be solved with QA adapting and extending methods proposed in previous studies [25,26]. Note that our aim is *not* to develop a solution method for the differential equation which is faster today than standard methods, but to explore QA with a concrete example relevant to physical oceanography and meteorology.

To utilize QA, we cast our differential equation to an equivalent optimization problem. As a first step, we choose the Stommel problem as an example. The Stommel model describes the large-scale wind-driven ocean circulation. Details are found in standard textbooks such as [27,28]. We test two methods. In one suggested by [25], we discretize the partial differential equation using finite differences. We can then solve the resultant linear equation using the standard least-squares method, which is an optimization problem. A previous study [25] tested this method but they had to use a large number of qubits to approximate real numbers, which makes it difficult to solve large problems like the discretized Stommel equation. Indeed, their solutions included significant errors even for a small problem using only 5 grid points. In this study, we show that the QA methods fail on modestly large problems and that an iteration procedure suggested by [26,29] is necessary. The other method we test is truncated spectral expansion [26], which approximates the solution by a truncated orthogonal basis series. For a large problem, this method also fails. We show that the latter method works only for an even smaller problem than the former method.

Our paper is structured as follows: We outline the quantum annealing method and its classical counterpart, “simulated annealing” (SA), in Section 2. We also reformulate a class of general linear partial differential equations as an optimization problem by the least squares method. In Section 3, we solve the Stommel problem and compare the outcomes of QA with those of SA. We also compare the performance of the iteration method with the direct binary expansion with large qubit numbers. Section 4 first summarizes the results and then compares our method with other potential quantum-computing methods.

## 2. Method

In this section, we first describe a general linear partial differential equation (Section 2.1) and discretize and cast it to a minimization problem in two ways (Section 2.2). We then formulate the (mathematical) minimization problem as energy minimization of an Ising model (Section 2.3) and explain how to find the minimal energy state (Section 2.4), which gives the solution to the original discretized differential equation. Since, as will be found in Section 3.2, it is often difficult to find the minimal energy state if the model uses too many variables at a time, we adopt an iteration method to minimize the size of the Ising model (Section 2.5). Finally, we describe how to “program” the actual physical machine, which acts as a programable Ising model, discussing the problem of “graph embedding” (Section 2.6).

### 2.1 Linear partial differential equations

In this study, we consider an order-m linear partial differential equation of the form


$${\;L[f]|}_{x\in X}\;:=\sum_{{\left|k\right|}_{mul}\leq m}C_{k}\left(x\right)\partial^{k}f\left(x\right)+B\left(x\right)=0,\;$$ (1)

where x=(x,y) is the position vector in ${\mathbb{R}}^{2}$, f is a real-valued function of x or a map from X→R, where X is a subset of ${\mathbb{R}}^{2}$ as shown below, $k=(k_{1},k_{2})$ is a multi-index of non-negative integers, ${\left|k\right|}_{mul}:=k_{1}+k_{2}$, $C_{k}$ is a real-valued variable coefficient, $\partial^{k}f:=\partial_{x}^{k_{1}}\partial_{y}^{k_{2}}f$, where $\partial_{x}^{k_{1}}$ and $\partial_{y}^{k_{2}}$ are the $k_{1}$-th and $k_{2}$-th derivatives with respect to x and y, and B is a real-valued inhomogeneous term. Here, we define the domain as


$$\begin{array}{c} X=\left\{x=(x,y)\;|\;x\in [0,1],y\in [0,1]\right\}. \end{array}$$ (2)

We restrict our attention in this paper to the case where Equation (1) admits a unique solution. To obtain an approximate solution to Equation (1), we minimize the following Hamiltonian or “cost function” [26,30]:


$$\begin{array}{c} H={\left\|L[f]\right\|}^{2}:=\int_{0}^{1}\int_{0}^{1}{\left|L[f](x,y)\right|}^{2}\;dxdy.\; \end{array}$$ (3)

We do not specify boundary conditions here for simplicity; our argument below holds as long as the boundary conditions are linear. Section 3 shows an example of Equation (1) with boundary conditions specified.

### 2.2 Discretization of the differential equation

To find a function *f* that minimizes the Equation (1) numerically, we discretize Equation (3) in two ways. One approach is to approximate the Equation (3) with finite differencing, representing f(x) with gridded values $w_{ij}=f(x_{i},y_{j})$, where $\left(x_{i},y_{j}\right)$ is a regular grid point in X; and the other approach is to expand f(x) with a truncated set of basis functions and express *H* in terms of the expansion coefficients.

#### 2.2.1 Finite differencing method.

If we discretize the domain *X* into $M^{\prime }=M\times M$ regular grid points ${\left\{\left(x_{i},y_{j}\right)\right\}}_{i,j\in \{1,2,\ldots ,M\}}$ and replace partial derivatives in Equation (1) with finite differences [31], the partial differential equation (1) together with the boundary conditions is converted to a set of linear equations


$$\begin{array}{c} Aw-v=0,\;\# \end{array}$$ (4)

where $A={\left\{a_{ij}\right\}}_{i,j\in \{1,2,\ldots ,M^{\prime }\}}$ is an $M^{\prime }\times M^{\prime }$ matrix, $w={\left\{w_{i}\right\}}_{i\in \{1,2,\ldots ,M^{\prime }\}}$ is the solution we seek, and $v={\left\{v_{i}\right\}}_{i\in \{1,2,\ldots ,M^{\prime }\}}$ is the vector representing the inhomogeneous term. There are various finite-difference methods; in Section 3, we choose one simple method for our differential equation. A least squares method forms the optimization problem


$$\begin{array}{c} w=\mathrm{\backslash arg}\min_{\hat{w}}{\left\|A\hat{w}-v\right\|}^{2}=\mathrm{\backslash arg}\min_{\hat{w}}H(\hat{w}), \end{array}$$ (5)

where


$$H\left(w\right):={\left\|Aw-v\right\|}^{2}=\sum_{i,j=1}^{M^{\prime }}J_{ij}w_{i}w_{j}+\sum_{i=1}^{M^{\prime }}h_{i}w_{i}+\sum_{i=1}^{M^{\prime }}v_{i}^{2},$$ (6)

$$J_{ij}=\sum_{k=1}^{M^{\prime }}a_{ki}a_{kj},$$ (7)

and


$$h_{i}=\sum_{j=1}^{M^{\prime }}\left(-2a_{ji}v_{j}\right).\;$$ (8)

Since the last term of Equation (6) is constant, it does not influence the optimization. In this way, **w** that minimizes (6) is an exact solution to (4). The error in the original problem, therefore, ultimately comes from the inaccuracy in the finite-difference discretization if this minimization problem is accurately solved.

#### 2.2.2 Truncated spectral expansion algorithm.

In the other method to discretize (3), we express the function *f* as an expansion in terms of a truncated basis consisting of real-valued functions, ${\left\{\varphi_{i}\right\}}_{i\in \{1,2,\ldots ,n_{basis}\}}$, as


$$f(x)=\sum_{i=1}^{n_{basis}}w_{i}\varphi_{i}(x),\;$$ (9)

where $n_{basis}$ is the order of expansion and $w_{i}$ is the real-valued expansion coefficient. (See [26], for example.) Then Equation (1) is written as


$$L[f](x)\approx \sum_{i=1}^{n_{basis}}w_{i}G_{i}(x)+B\left(x\right),$$ (10)

where $G_{i}$ is defined as


$$G_{i}(x)=\sum_{{\left|k\right|}_{mul}\leq m}C_{k}(x)\partial^{k}\varphi_{i}(x).$$ (11)

For the function f to be an approximate solution to (1), the cost function below should be minimized:


f (12)

$$=\sum_{i,j=1}^{n_{basis}}J_{ij}w_{i}w_{j}+\sum_{i=1}^{n_{basis}}h_{i}w_{i}+\int_{0}^{1}\int_{0}^{1}{\left|B(x)\right|}^{2}dxdy,\;$$ (13)

where


$$\begin{array}{c} J_{i,j}=\int_{0}^{1}\int_{0}^{1}G_{i}(x)G_{j}(x)\;dxdy,\; \end{array}$$ (14)

$$\begin{array}{c} h_{i}=2\int_{0}^{1}\int_{0}^{1}B\left(x\right)G_{i}(x)\;dxdy. \end{array}$$ (15)

The solution to the original problem is again converted to the minimization of a cost function. In the previous subsection (finite differencing), the conversion from *f*(**x**) to $w={\left\{w_{i}\right\}}_{i\in \{1,2,\ldots ,n_{basis}\}}$ happened by finite differencing, and in the present subsection (truncated spectral expansion), the conversion happens by spectral expansion. The accuracy of the solution to the original differential equation therefore strongly depends on the selected basis and the degree of truncation. A previous study [26] confirmed its efficacy in solving simple partial differential equations whose solutions can be well represented by a limited number of basis functions.

It should be noted that the expression (9) is not an exact solution to the original differential equation; it is merely an approximate solution, and its accuracy inherently depends on where we truncate the spectral expansion. For this reason, the cost function does not approach zero. In contrast, the finite difference form (Equation (4)) has an exact solution, for which the finite-difference cost function vanishes. The spectral expansion method, however, has the advantage of being applicable to nonlinear equations (Appendix 1 in S1 Text).

### 2.3 Transformation of cost function into an Ising model

We describe basic principles of the Ising model and its connection to the minimization problem discussed in Section 2.2. The Ising model was originally proposed as a model to understand ferromagnetism. It consists of *N* spins located at lattice sites. Each spin can exist in one of two states: “upward” or “downward.” The Ising Hamiltonian, representing the energy of the system, is given by


$$H_{0}=-\sum_{i,j\in V}{J_{ij}\sigma }_{i}\sigma_{j}-\sum_{i\in V}h_{i}\sigma_{i},\;$$ (16)

where V={1,2,...,N} is the set of the spin sites*,* $\sigma_{i}$ is a *spin variable*, a type of binary variable that can take 1 or −1, $J={\left\{J_{ij}\right\}}_{i,j}$ is a real-valued *N × N* symmetric matrix representing the interaction between the *i*-th and *j*-th spins, and $h_{i}\;$is the real-valued external field that acts on the *i*-th spin. The state $\sigma =\{\sigma_{1},\;.\;.\;.\;,\sigma_{N}\}$ that minimizes the Hamiltonian is known as the *ground state*. In general, ground states can be degenerate (i.e., different states having the same energy value), but for the problems we deal with in this manuscript, the ground states are not degenerate if the accuracy of the approximation is sufficient because the solution is uniquely determined.

The form of cost functions introduced in Section 2.2 (Equations (6) and (13)) is identical to that of the Ising Hamiltonian (Equation (16)) except for whether the variables are real or binary and except for the constant term. To transform the cost functions to the Ising Hamiltonian, the real-valued variables, w of Equations (6) and (13), need to be approximated by several spin variables. We first expand w in terms of spin variables σ as


$$w_{i}=c_{i}+s_{i}\sum_{\alpha =0}^{n_{spin}-1}\frac{\sigma_{i}^{\left(\alpha \right)}}{2^{\alpha }},$$ (17)

where $\left\{\sigma_{i}^{\left(0\right)},\sigma_{i}^{\left(1\right)},\;.\;.\;.\;,\;\sigma_{i}^{{(n}_{spin}-1)}\right\}$ are the spin variables used to represent the single real value $w_{i}$ and $n_{spin}$ is the number of spins per variable, $s_{i}$ is a real-valued *scale factor* and $c_{i}$ is a real-valued *control parameter*. We determine the scale factor and control parameter by the iterative process named “zooming extension” [29] (see Section 2.5 in detail). This is an extension of the usual binary fractional representation of a real number: $w\;=\;c\;+\;n_{0}/2^{0}\;+\;n_{1}/2^{1}\;+\;n_{2}/2^{2}\;+\;.\;.\;.\;=\;c\;+\;n_{0}.n_{1}n_{2}.\;.\;.$ in binary fraction, where *n*’s are either 0 or 1. Substituting the binary expansion (Equation (17)) to the cost function (See Equations (6) or (13)), we obtain an Ising Hamiltonian. If the scale factor and control parameter are appropriately chosen, the spin variables of the ground state give the global minimum of the cost function up to a precision of $s_{i}2^{-n_{spin}}$. It should be noted that if $w_{i}$ is directly binary-expanded, the necessary spin number increases [25] with solutions more often failing as discussed in Section 3.4. The above “zooming extension” enables to increase the chance of success with small spin numbers as discussed in Section 2.5.

### 2.4 Annealing method

“Annealing” is originally a heat treatment of a material such as steel, but the term is borrowed from metallurgy for a general algorithm that searches for the ground state of an Ising model or cost function, whereas there are specialized algorithms for special cases. It is noted that one cannot tell in general whether annealing finds the ground state for each case. In this sense, annealing is a semi-empirical method. We introduce two types of annealing: simulated annealing (SA) and quantum annealing (QA).

#### 2.4.1 Simulated annealing.

SA is a probabilistic approach. We investigate the characteristics of the SA approach as the classical counterpart of QA in this study. (SA itself has been used for oceanographical optimization problems outside the context of quantum annealing [32–34].)

During each iteration of SA, a single spin, chosen at random, is inverted. If this change results in a reduction of the Hamiltonian, the new spin configuration is accepted. Conversely, if the Hamiltonian increases, the new configuration may still be accepted, albeit with a less-than-one probability. The Metropolis algorithm, as shown in [35], is frequently employed to determine this probability of acceptance:


$$\begin{array}{c} P(\Delta E,T)=\left\{ \begin{array}{c} 1,\;\;\Delta E\leq 0\\ \exp\left(-\frac{\Delta E}{k_{B}T}\right),\;\;\Delta E>0 \end{array}\right.\;\;\;\;\;\;\;\;\;,\; \end{array}$$ (18)

where T is the “temperature”, $k_{B}$ is the Boltzmann constant, and ΔE is the energy difference of the new configuration from that of the old configuration. It is noteworthy that the second row is a general probability of thermal fluctuation called the Boltzmann distribution; thus, SA conducts a ground state search through simulated thermal fluctuation, just as the physical annealing of metal or alloy brings it to a lower-energy state. If the system is trapped in a local minimum, these fluctuations facilitate an escape. This allows the continued pursuit of the global minimum. This “hill-climbing” feature can be a significant advantage over gradient descent algorithms [35].

We repeat the Metropolis algorithm by decreasing the temperature in each step, which is equivalent to the reduction of the probability of accepting worse solutions. If the schedule of the temperature decrease is sufficiently slow in such a way that


$$\begin{array}{c} T(t)\geq \frac{aN}{\log(\alpha t+2)},\; \end{array}$$ (19)

the error between the solution obtained from SA and the true solution falls within a desired acceptable range [35,36]. Here, a and α are some constants. This condition shows that the time for T(t) to reach a sufficiently small value ∈ is


$$\begin{array}{c} t\sim \exp\left(\frac{N}{\in }\right), \end{array}$$ (20)

indicating that the temperature schedule should be exponentially slower for a larger number of spins. It is noteworthy that the condition is not an optimal estimate but just the worst-case estimate.

#### 2.4.2. Quantum annealing.

The QA approach is also an algorithm that searches for the ground state of the Ising model. While SA utilizes simulated thermal fluctuations, quantum fluctuations are responsible for the search in QA [12]. We provide a brief introduction to QA in this section, and Appendix 2 in S2 Text provides an intuitive introduction to QA for readers unfamiliar with the quantum mechanism. We describe the procedure for the D-Wave machine. Some specific details may be different on different machines.

We define a time-dependent Hamiltonian of the system as follows:


$$\begin{array}{c} \hat{H}(s)=A(s){\hat{H}}_{0}+B(s){\hat{H}}_{i},\; \end{array}$$ (21)

where ${\hat{H}}_{0}$ is the quantum Ising Hamiltonian, ${\hat{H}}_{i}$ is an initial Hamiltonian, and A(s) and B(s) are weight functions [11,12,14]. The scaled time s = t / Δt is referred to as an annealing schedule; s = 0 at the initial time t = 0 and s = 1 at the final time t = Δt. Throughout this work, we fix Δt = 20 μs unless otherwise stated. The weight function A(s) monotonically increases from A(0= 0 to A(1= 1, and B(s) monotonically decreases from B(1= 1 to B(0= 0. To differentiate H from the classical Hamiltonian, the quantum version is denoted with a hat symbol. In the context of QA, a “transverse field” is often selected as the initial Hamiltonian since its ground state is known and easily prepared. This field drives the quantum fluctuation and hence corresponds to the thermal fluctuation of SA. If the state consistently stays in the ground state of $\hat{H}(s)$ at all times, it will naturally converge as s →1 to the ground state of ${\hat{H}}_{0}$, which is the solution we seek, because A(s)→1 and B(s)→0.

The *adiabatic theorem* [37] ensures this expectation, provided changes in $\Gamma (t):=d\hat{H}\;/dt$ is sufficiently slow. According to [36,38], a sufficient condition for convergence of QA is


$$\begin{array}{c} \Gamma (t)\geq r(\delta t+c{)}^{-1/(2N+1)},\; \end{array}$$ (22)

where r and c are constants, δ is a small parameter, and N is the number of spin variables. This condition shows that the time for Γ(t) to reach a sufficiently small value ∈ is


$$\begin{array}{c} t\;\sim \exp\left(N\left|\log\in \right|\right). \end{array}$$ (23)

This condition is not an optimal estimate but a worst-case one as in the case of SA. A comparison of Equations (20) and (23) shows that both SA and QA take exponential time to converge at worst, but the QA approach is better than the SA approach since $\left|\log\in \right|\ll \in^{-1}$ for 0≤∈≪1.

### 2.5 Implementation of annealing for practical problems

To obtain a solution, which is a set of real numbers for us, on an annealing machine, which uses binary variables (spins), there are two methods. One is to express each real number as a series of binary variables (Equation (17) with $s_{i}=1$) [25]. If there are $M^{\prime }$ real values to solve for, we need $N\;=\;M^{\prime }n_{spin}$ spin variables. Although straightforward, this approach reduces the likelihood of attaining the global minimum due to increased spin numbers. In addition, the required annealing time increases exponentially with N at worst (Equations (20) and (23)).

Alternatively, we use an iteration procedure named “zooming extension” [26,29] with a small $n_{spin}$. In each iteration *I*, which we call an *epoch*, the hyperparameters of Equation (17) are updated as follows:


$$\begin{array}{c} c_{i}^{\left(I\right)}=w_{i}^{\left(I-1\right)},\; \end{array}$$ (24)

$$\begin{array}{c} s_{i}^{\left(I\right)}=Ss_{i}^{\left(I-1\right)}, \end{array}$$ (25)

where S is a scale factor to “zoom in” in each epoch. Simply put, we initially obtain a very crude approximate solution with $w_{i}\;=\;\pm 1$. Next, we look for a better approximation around this initial solution, narrowing the range of the search by a factor of 1/*S* and rewriting *H* in terms of the new $w_{i}$. We repeat this “zooming extension” until we arrive at the desired precision. A notable potential drawback is that if incorrect values are obtained for $w_{i}$ in an earlier iteration, they cannot be corrected in the subsequent iterations unless the scale factor *S* is close to 1 but the convergence is slower then. We will discuss the impact of the parameter *S* on accuracy in Section 3.

### 2.6 Programming an annealing machine

We implement our SA code using *Fixstars Amplify SDK*, a Python library for formulating combinational optimization problems [39]. The SDK provides data structures for SA and QA and functions to operate on them. Spins of SA are fully connected; that is, $J_{ij}$ can be nonzero for any pair of (*i,j*). The annealing schedule is automatically optimized by the SDK in this study.

For QA, we use the D-Wave annealer *Advantage_system4.1.* Because of physical noise, identical annealing runs give different results. For this reason, we run QA 500 times per iteration and choose the best solution, that is, the one that gives the smallest Hamiltonian value. Unlike SA, the D-Wave annealer has limited connectivity, that is, $J_{ij}\;$has to be zero for a large number of (*i,j*) pairs; in the Ising-model terminology, not all pairs of spins can interact. Only “neighbors” can interact, and “long-range” interactions do not exist. This is a serious problem because, for example, spectral expansion naturally includes long-range interactions.

**Fig 1:**
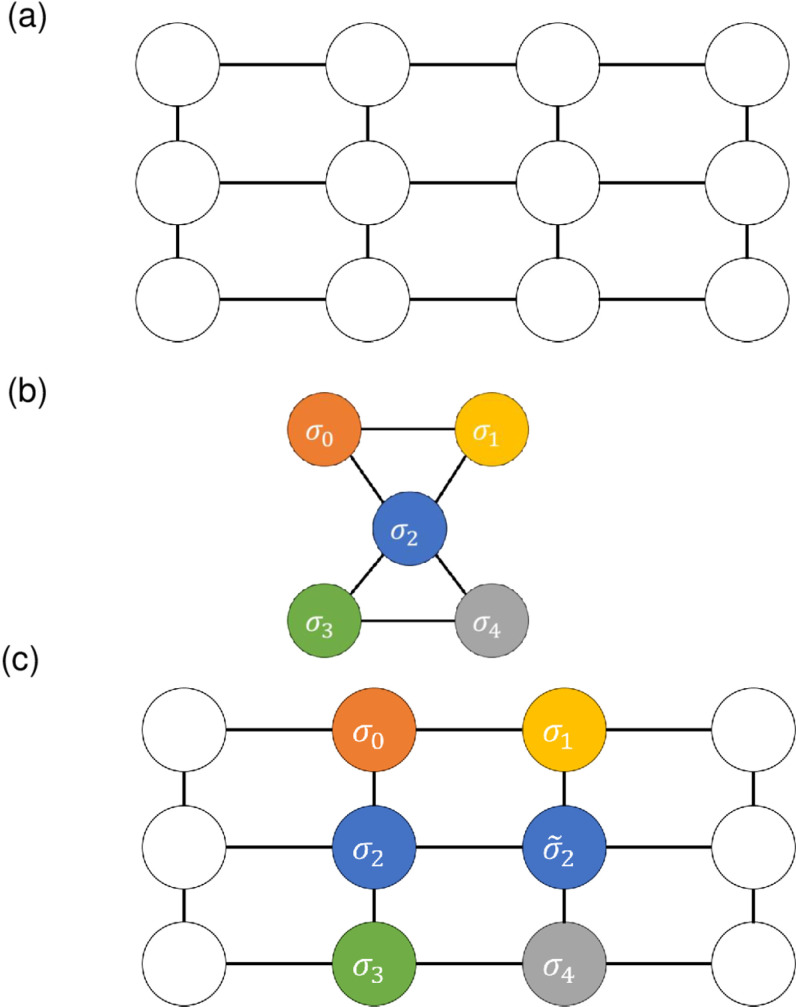
Schematics for graph embedding. Schematics showing (a) the spin configuration of a virtual annealing machine, (b) the spin configuration of the Hamiltonian (Equation (26)), and (c) how the Hamiltonian is embedded in the machine’s connectivity graph. The blue-colored qubits $\sigma_{2}$ and ${\tilde{\sigma }}_{2}$ are introduced to represent the graph topoglogy of (b). A penalty term will be added to the Hamiltonian to enforce the equality of $\sigma_{2}$ and ${\tilde{\sigma }}_{2}$.


To go around this limitation, the Hamiltonian is transformed by embedding the connectivity graph of $J_{ij}$ in the graph of the hardware [40]. (D-Wave forms what is called a Pegasus graph.) To illustrate graph embedding, we consider, instead of the actual D-Wave machine, a simple toy machine whose spin connectivity graph is represented by Fig 1(a) (this example follows [39]). For illustration purposes again, let us assume that our problem has this Hamiltonian:

$$\begin{array}{c} H=\sigma_{0}\sigma_{1}+2\sigma_{1}\sigma_{2}-3\sigma_{0}\sigma_{2}+4\sigma_{2}\sigma_{3}-5\sigma_{3}\sigma_{4}-6\sigma_{2}\sigma_{4}. \end{array}$$ (26)

**Fig 2:**
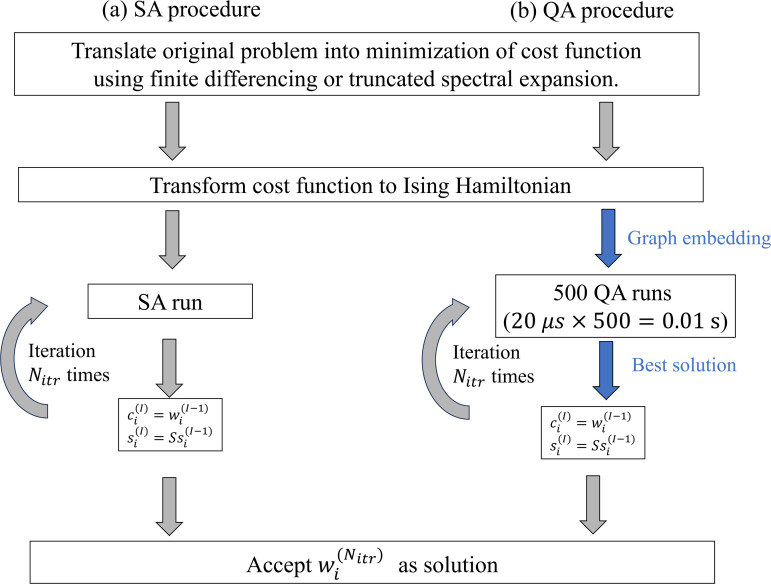
Schematics of SA and QA procedure. Schematic of procedure of (a) SA and (b) QA approaches. The parameters $c_{i}\;$and $s_{i}$ are the control parameter and scale factor of the Equation (17). We iterate the SA runs $N^{\left(itr\right)}$ times to determine the control parameter and scale factor. In QA, we conduct 500 times of QA runs with the annealing time of 20 μs, and iterate this procedure $N^{\left(itr\right)}$ times.

The connectivity of the Hamiltonian is shown in Fig 1(b); obviously, this connectivity cannot be directly represented by the machine’s native graph. To represent this connectivity in the graph of the machine (Fig 1(a)), two spin variables of the machine are assigned to represent the single spin $\sigma_{2}$ of the Hamiltonian as shown in Fig 1(c) (The additional spin is named ${\tilde{\sigma }}_{2}$). To ensure that the two spin variables have the same value, a penalty term ($J_{2,\tilde{2}}\;\sigma_{2}\;{\tilde{\sigma }}_{2}$ with $J_{2,\tilde{2}}<0$ to penalize the cases where $\left(\sigma_{2},\;{\tilde{\sigma }}_{2}=\;(1,\;-1)\;or\;(-1,\;1\right)$) is added to the Hamiltonian. The strength of the penalty term $\left|J_{2,\tilde{2}}\;\sigma_{2}\;{\tilde{\sigma }}_{2}\right|$ must be large enough to ensure that the two spin variables are the same but small enough not to dominate the optimization and lead to a solution far from the ground state of the original Hamiltonian (When the penalty term is too small and $\sigma_{2}$ and ${\tilde{\sigma }}_{2}$ take different values, this is referred to as a *chain break*.). This trade-off can sometimes be difficult [41]. For this and other reasons, graph embedding by itself is a subject of active research. In this paper, we use a library in “Fixstars Amplify SDK” for this transformation of the Hamiltonian [At first, the clique embedding [42] is tried, and the minorminer embedding [43] is conducted if the clique fails. In the library, the strength of the penalty is determined by uniform torque compensation (https://docs.ocean.dwavesys.com/projects/system/en/latest/reference/generated/dwave.embedding.chain_strength.uniform_torque_compensation.html)]. The broken chains are determined by the majority rule. Fig 2 summarizes the procedures of SA and QA.

## 3. The Stommel problem

**Fig 3:**
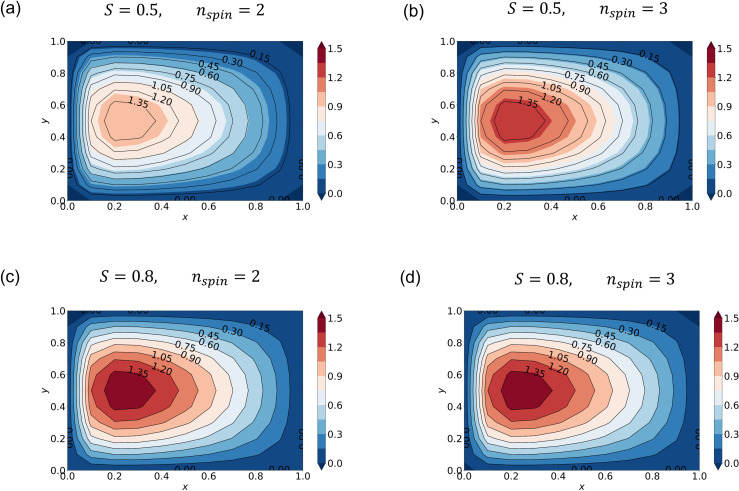
Streamfunction calculated by SA with different hyperparameters. Streamfunction averaged over 10 runs from SA (shading) and the “true” solution (black contour lines) with M=11 and ∊ = 0.1. The true solution is obtained with standard linear algebra. The hyperparameters are (a) $\left(S,n_{spin})=(0.5,\;2\right)$, (b) $\left(S,n_{spin})=(0.5,\;3\right)$, (c) $\left(S,n_{spin})=(0.8,\;2\right)$, and (d) $\left(S,n_{spin})=(0.8,\;3\right)$.

In the last section, we described how one would solve an abstract partial differential equation of the form (1) using annealing methods. In this section, we use the “Stommel problem” as an example of (1), comparing finite difference with truncated spectral expansion, SA with QA, and iteration with spin series. The basin-scale oceanic circulation in mid-latitudes is largely determined by large-scale wind stress and can be described by vertically integrated horizontal velocity of water flow. Since the vertically integrated horizontal velocity is nearly divergence-free, it can be described by (−∂ψ/∂x, ∂ψ/∂y), where the function ψ(x,y) is called the “stream function” as the velocity is parallel to the local isopleth of ψ and its strength is proportional to its gradient. According to [27,28], the streamfunction is a solution to the partial differential equation


$$\begin{array}{c} \frac{\partial \psi }{\partial x}+\in \left(\frac{\partial^{2}\psi }{\partial x^{2}}+\frac{\partial^{2}\psi }{\partial y^{2}}\right)+\frac{\partial \tau_{x}}{\partial y}=0, \end{array}$$ (27)

where ψ is the streamfunction, x∈[0,1] and y∈[0,1] are the eastward and northward coordinates, $\tau_{x}=-\cos\left(\pi y\right)$ is wind stress, which is prescribed, and ∈ is a small parameter representing friction. We assume that ψ=0 at the lateral boundaries; since the direction of the depth-integrated velocity is parallel to the isopleth of ψ, this boundary condition is a consequence of the fact that there is no flow across the boundaries. Here, all variables are nondimensionalized and we set ∈=0.1 as an example unless otherwise stated. For the real ocean, ∈ (friction) is determined by nature and cannot be set arbitrarily, but for the present study, we set it to convenient values; ∈ is proportional to the width of the narrow flow near the left edge of the domain (see Fig 3 below, for example) and so a smaller value requires higher resolution in x when the problem is discretized as below. The uniqueness of the solution for Equation (27) is shown in Appendix 3 in S3 Text.

To identify Equation (27) with Equation (1), we recognize that


$$\begin{array}{c} L[f](x,y)=\in \left(\frac{\partial^{2}}{\partial x^{2}}+\frac{\partial^{2}}{\partial y^{2}}\right)f(x,y)+\frac{\partial }{\partial x}f(x,y)+\frac{\partial \tau_{x}}{\partial y}. \end{array}$$ (28)

### 3.1. Finite-difference solution using SA

We discretize the domain into an $M^{\prime }=M\times M$ regular grid with M=11. The streamfunction and wind stress are discretized as ${\left\{\psi_{i,j}\right\}}_{i,j\in \{1,2,\ldots ,M\}}$ and ${\left\{\tau_{i,j}\right\}}_{i,j\in \{1,2,\ldots ,M\}}$, respectively. Using the central differencing, Equation (27) is discretized as


$$\begin{array}{c} \frac{\psi_{i+1,j}-\psi_{i-1,j}}{2\Delta x}+\in \left(\frac{\psi_{i+1,j}-2\psi_{i,j}+\psi_{i-1,j}}{\Delta x^{2}}+\frac{\psi_{i,j+1}-2\psi_{i,j}+\psi_{i,j-1}}{\Delta y^{2}}\right)=u_{i,j},\; \end{array}$$ (29a)

where


$$u_{i,j}=-\frac{\tau_{i,j+1}-\tau_{i,j-1}}{2\Delta y},$$

and the boundary condition are


$$\begin{array}{c} \psi_{1,j}=\psi_{M,j}=0\;\;\;\;\;\;\;\;\;\;\;\;for\;j=1,\ldots ,M,\; \end{array}$$ (29b)

$$\begin{array}{c} \psi_{i,1}=\psi_{i,M}=0\;\;\;\;\;\;\;\;\;\;\;\;for\;i=1,\ldots ,M. \end{array}$$ (29c)

Here, Δx=Δy=1/(N−1) represent the grid spacing in the east-west and north-south directions, respectively. In total, the equation set (29a) includes $(M-2{)}^{2}+2M+2M-4=M^{2}=M^{\prime }$ equations for the $M^{\prime }$ values ${\left\{\psi_{i,j}\right\}}_{i,j\in \{1,2\ldots ,M\}}$.

Next, we arrange the grid point values ${\left\{\psi_{i,j}\right\}}_{i,j\in \{1,2\ldots ,M\}}$ into a vector


$$\begin{array}{c} w={\left(\psi_{1,1},\psi_{2,1},..,\psi_{M,M}\right)}^{T}, \end{array}$$ (30)

where $\cdot^{T}$ indicates the transpose of a vector. The wind stress terms and the right-hand sides (zeros) of the boundary conditions are correspondingly arranged into a vector


$$\begin{array}{c} v^{T}=\left(0,\ldots ,0,u_{2,2},u_{3,2},u_{4,2},\ldots ,u_{M-1,M-1},0,\ldots ,0\right),\; \end{array}$$ (31)

where $u^{\prime }s$ are in the rows corresponding to Equation (29a) and $0^{\prime }s$ are in the rows corresponding to the boundary conditions. The set of equations (29a) is then written as


$$\begin{array}{c} Aw=v,\; \end{array}$$ (32)

where A is the sparse matrix of the coefficient in (29a), whose non-zero components involving 1/2Δx, $\in /\Delta x^{2}$, and $\in /\Delta y^{2}$ as well as 1 for the rows corresponding to the boundary conditions.

As in Section 2.2.1, we solve Equation (32) using a least-squares minimization of a cost function. We also solve it using standard linear algebra. We refer to this solution as “true” (because (32) has an exact solution that can be obtained with standard linear algebra) and compare it with solutions from the annealing methods. The linear algebra calculation implemented using the LAPACK library (through the Numpy library of Python [44]) takes ~0.01 s on the lead author’s desktop PC [Windows 11, 2.4GHz, 64 GB, 20 cores], which corresponds to one iteration time of QA (see section 2.6) on the actual D-Wave machine. At this point, annealing is not faster than classical linear algebra on actual machines for our problem at hand.

**Fig 4:**
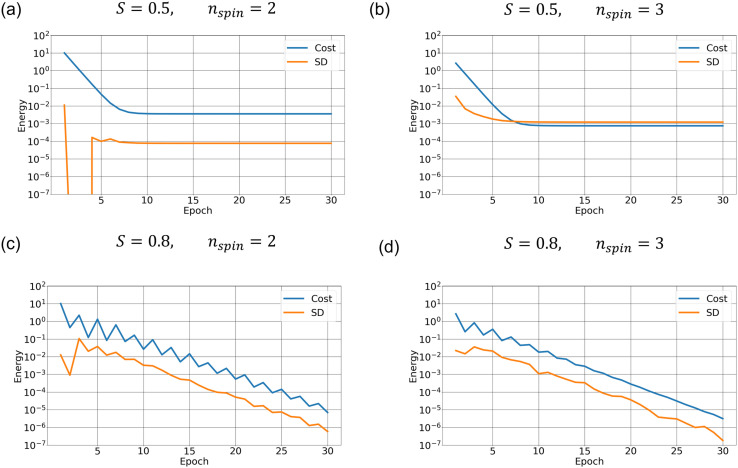
Cost function calculated by SA with different hyperparameters. Cost function (blue curve) and its standard deviation (orange curve) versus epoch calculated over 10 runs for the same four experiments as in Fig 3. The hyperparameters are (a) $\left(S,n_{spin})=(0.5,\;2\right)$, (b) $\left(S,n_{spin})=(0.5,\;3\right)$, (c) $\left(S,n_{spin})=(0.8,\;2\right)$, and (d) $\left(S,n_{spin})=(0.8,\;3\right)$.

To investigate how the annealing solution depends on *S* and $n_{spin}$ (Section 2.5), we conduct SA experiments with $\left(S,n_{spin})=(0.5,\;2\right)$, $\left(S,n_{spin})=(0.5,\;3\right)$, $\left(S,n_{spin})=(0.8,\;2\right)$, and $\left(S,n_{spin})=(0.8,\;3\right)$. Given the stochastic nature of annealing, we performed each experiment 10 times in order to estimate the variability in the convergence behavior of the cost function. Fig 3 and Fig 4 plot the mean streamfunction and cost function calculated over the 10 runs. Stochastic variability in cost function, defined as the standard deviation at each epoch, is also plotted. The black contours in Fig 3 indicate the “true solution”. With $\left(S,n_{spin})=(0.5,\;2\right)$, the SA solution is significantly different from the true one (Fig 3a) even though it is well converged (Fig 4a). Note that the cost function converges to a much higher value (Fig 4a) than for the better solutions (Fig 4b-d), which indicates that this one has converged to an incorrect solution.

Increasing the number of spins improves accuracy. When $n_{spin}=3$, the cost function (Fig 4b) becomes smaller and the solution is improved (Fig 3b). However, the magnitude of stochastic variability in the cost function is comparable to that of the cost function itself, indicating a lack of sufficient convergence. Setting *S* = 0.8, by contrast, gives a better solution even with $n_{spin}\;=\;2$ (Fig 3c). The convergence is much slower (Fig 4c) and the accuracy still keeps improving after 30 epochs. In this case, the stochastic variability is approximately one order of magnitude smaller than the cost function value at each epoch. Increasing the number of spins from 2 to 3 again does not give a significant improvement (Fig 4c and Fig 4d). This is a natural result because increasing $n_{spin}$ has effectively the same impact as increasing the number of iterations (Section 2.5) as long as both converge to the same solution. Indeed, $n_{spin}\;=\;3$ converges somewhat faster than $n_{spin}\;=\;2$ (Fig 4c and Fig 4d).

It should be noted that S and iteration step numbers should be adjusted following the spin number. If $n_{spin}=1$, it takes more than 120 iteration steps with S=0.95 (not shown). It is also confirmed that the SA procedure with $n_{spin}=1$ results in wrong solutions if S remains 0.8. This is useful information for future extensions of this method.

### 3.2. QA solution to the finite-difference form

**Fig 5:**
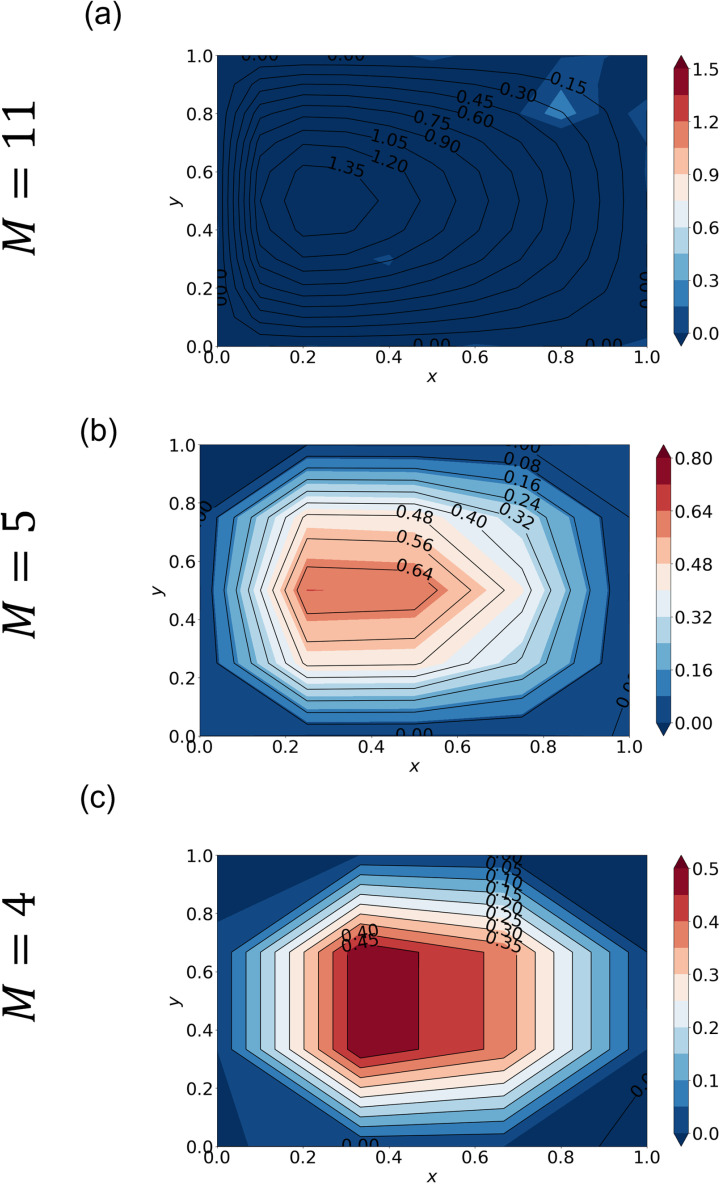
Streamfunction calculated by QA with different with different grid resolutions Streamfunction averaged over 10 runs from QA (shading) and the “true” solution (black contour lines) for (a) M=11 and ∈=0.1, (b) M=5 and ∈=0.25, and (c) M=4 and ∈=1/3. The hyperparameters are $\left(S,n_{spin})=(0.8,\;2\right)$ for all cases. Noting that the cost function of QA with $M^{\prime }=121$ remains on the order of 1.0 after 30 epoch, while it is on the order of $10^{-5}$ in the SA case of $\left(S,n_{spin})=(0.8,\;2\right)$.

**Fig 6:**
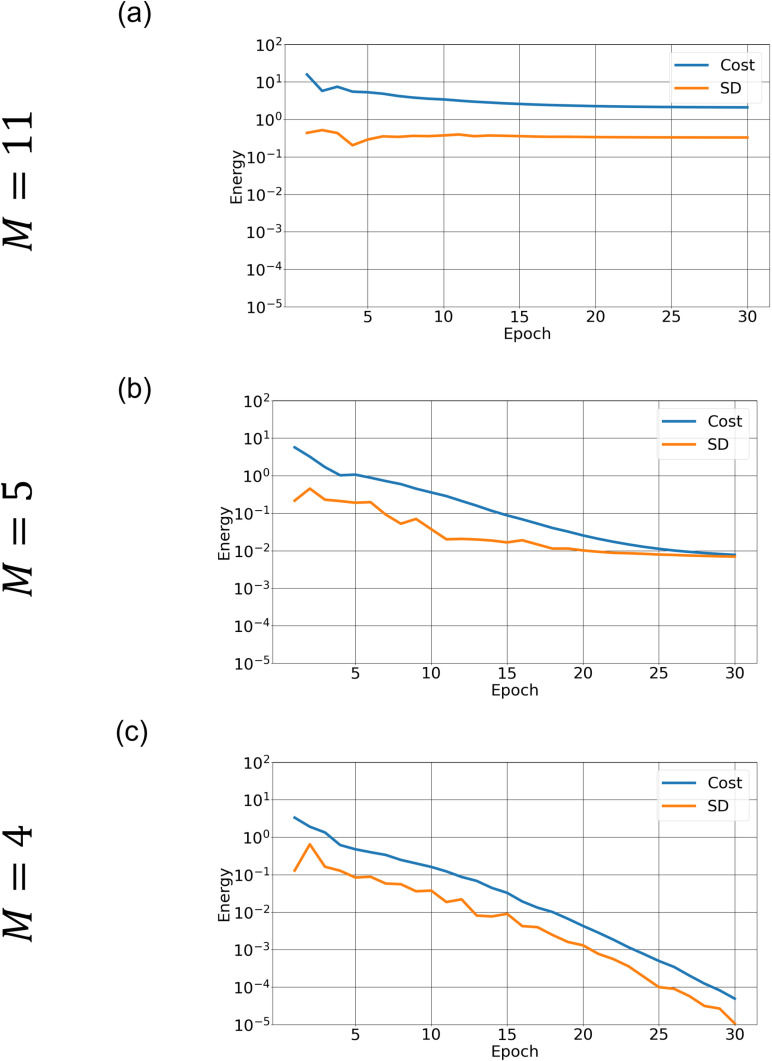
Cost function calculated by QA with different hyperparameters. Cost function (blue curve) and standard deviation (orange curve) versus epoch calculated over 10 runs for the same three experiments as in Fig 5. The hyper parameters are (a) M=11 and ∈=0.1, (b) M=5 and ∈=0.25, and (c) M=4 and ∈=1/3 with $\left(S,n_{spin})=(0.8,\;2\right)$.

We solve the same problem using the D-Wave annealer. We only test the $\left(S,\;n_{spin}=\;(0.8,\;2\right)$ case. Fig 5(a) shows that the QA approach fails to solve it. Accordingly, the cost function remains larger than 1.0 (Fig 6(a)). There are several possible reasons for this failure. One is that the required annealing time is much larger than those we have tested because of the increased number of spin variables needed to embed the graph (Section 2.6). The cost function consists of $n_{spin}\times M^{\prime }=242$ spin variables, and when embedded into the Pegasus graph using the algorithm described in Section 2.6, it used approximately 1500 qubits. Even when the iteration time was increased to 500 µs, no improvement in the results was observed (not shown). The second potential reason is that solutions from the machine always include errors due to noise on the hardware [19]. Other possible error sources include the accumulation of errors when mapping Ising coefficients onto the D-Wave hardware. The coefficients $J_{ij}$ and $h_{i}$ are usually expressed in a 32-bit or 64-bit floating-point representation on usual classical computers today, but on D-Wave, they are not as accurately represented in D-Wave since the $J_{ij}$ and $h_{i}$ values in the hardware are physically set by spatially local magnetic fields (https://docs.dwavesys.com/docs/latest/c_qpu_ice.html).

It should be noted that the chain strength is not the cause of computational failure. Even in cases where the computation failed, no chain breaks were observed. Furthermore, reducing the chain strength determined by uniform torque compensation by a factor of 0.1 did not lead to any improvements in the results. These results suggest that the failure is unlikely to be due either to chain breaks or to the dominance of the penalty terms.

We next reduce the resolution to a 5 × 5 regular grid and widen the boundary current by increasing ∈ = 0.25. (Note that there is a strong northward flow near the western edge of the domain in Fig 3d.) In this case, approximately 250 qubits are used after performing the graph embedding. As shown in Fig 5(b) and Fig 6(b), the accuracy is dramatically improved, and the value of the cost function is below $10^{-1}$. The variability in cost function values, however, is comparable to their mean values. When the same problem is solved using SA, the cost function decreases to values below $10^{-5}$ (not shown), indicating that QA shows significantly poorer convergence than SA. This suggests that the sources of error discussed in the previous paragraph are also non-negligible in this case. Although we have not tried it in this study, postprocessing such as the greedy algorithm (https://docs.ocean.dwavesys.com/en/stable/examples/pp_greedy.html) will reduce the error [45]. When M is reduced to 4, the number of required qubits decreased to approximately 150, and the convergence significantly improves (Fig 5(c) and Fig 6(c)). In this case, the cost function decreases to values below $10^{-4}$, and the stochastic variability is approximately one order of magnitude smaller than the cost function value at each epoch, suggesting that convergence tends to improve as the number of qubits decreases.

In theory, all optimization problems of the form (21) can be solved if the annealing time is sufficiently increased (Section 2.4.2) but that is not necessarily true for the D-Wave machine. The above results therefore suggest that better graph embedding algorithms and better representations of the coefficients $J_{ij}$ and $h_{i}$ would improve performance even for large problems. Equation (27) is a two-dimensional elliptic partial differential equation and the matrix of its finite-difference version is a very sparse matrix whose graph can be significantly different from the machine’s native graph, necessitating good embedding algorithms. Given that the elliptic partial differential equation arises in important oceanographic and meteorological techniques (such as calculating wave energy flux [46] or Helmholtz decomposition for atmospheric circulations [47]), it would be worthwhile to implement a multi-dimensional elliptic partial differential equation solver using QA.

### 3.3. QA and SA solutions with truncated spectral expansion

We apply the truncated spectral expansion approach to the Stommel problem. Given that the streamfunction must vanish at the lateral boundaries, we approximate it using a low-order truncated Fourier sine expansion


$$\psi (x,y)=\sum_{n,m}w_{nm}\sin(\pi nx)\sin(\pi my),$$ (33)

where $w_{nm}$ are the real-valued expansion coefficients. The boundary condition that ψ = 0 at the boundaries is already satisfied. Here, we assume that $n\in \{1,2,\ldots ,n_{x}\}$ and m∈{1,2}; we use only 2 modes in the *y* direction because we know that the solution will be very smooth in the *y* direction, while we set $n_{x}=10$ because we know that the solution includes a higher-wavenumber structure near the left boundary when ∈ is small (Fig 3, for example). As described in Section 2.2.2, we plug (33) into (27) and follow the derivation in Section 2.2.2 to obtain the Hamiltonian to minimize. Here, we set $n_{spin}=2$. The solution to this minimization problem is the optimal set of *w*’s in (33).

**Fig 7:**
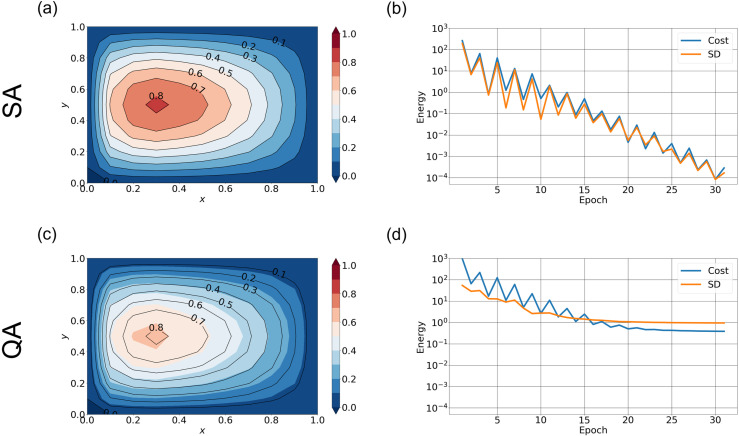
QA and SA solutions by truncated spectral expansion when $n_{x}=10$. (a) Streamfunction averaged over 10 runs from SA (shading) and CG (black contour lines). (b) The difference of cost function values between the SA and CG methods (blue curve) and its standard deviation (orange curve) versus epoch calculated over 10 runs. (c) and (d) are the same as the upper panels but for QA. The hyperparameters are $\left(S,n_{spin})=(0.8,\;2\right)$.

The SA method stably returns the global optimization when the hyperparameter S is sufficiently close to 1.0 (Section 3.1); we have confirmed the accuracy of SA solutions comparing them with those obtained by the conjugate gradient (CG) method on the cost function as a true solution. We terminate the iteration of CG when the gradient norm is decreased below $10^{-3}$. As shown in Fig 7(a), the streamfunction from SA is in agreement with that from the CG method. The difference in cost function values between the SA and CG methods decrease to $\sim 10^{-4}$ (Fig 7(b)), suggesting that the SA stably returns the true solution. Note that due to the truncation error of the spectral expansion, the cost function does not become zero even when each method converges to the global minimum. Therefore, we compare the differences between the two methods here.

Fig 7(c) shows the streamfunction obtained from QA. Although the QA solution reproduces the shape of the streamfunction of the true solution, its amplitude is smaller. Furthermore, the difference in cost function values between QA and CG methods (Fig 7(d)) is larger than $10^{-1}$, suggesting the poor performance of QA.

This failure is likely attributable to the number of spins, as in the case of the finite difference method. In the present case, the cost function is composed of 40 spins and embedding this problem into the annealing machine’s graph required approximately 200 qubits. Consequently, errors associated with embedding (Section 3.2) and hardware noise are likely to have been the cause of the failure.

**Fig 8:**
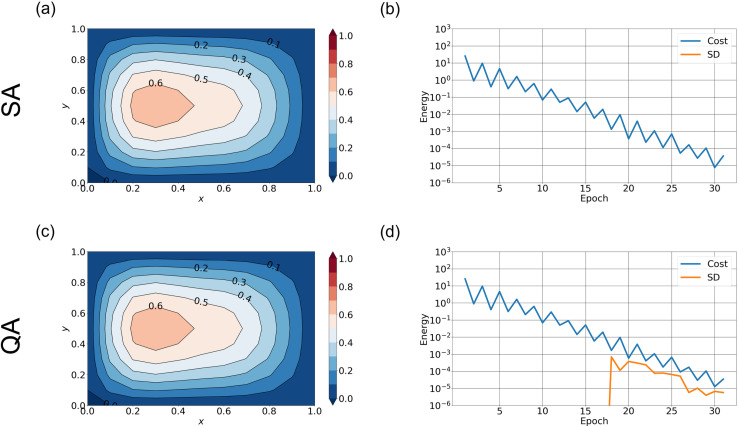
QA and SA solutions by truncated spectral expansion when $n_{x}=5$. Same as Fig 7 except for the number of zonal basis, $n_{x}$. The stochastic fluctuations smaller than $10^{-6}$ are beyond the resolution limit.

In the case where $n_{x}=5$, the performance of QA is significantly improved. The cost function consists of 20 spins and is represented using 60 qubits on the D-Wave annealer in this case. As shown in Fig 8(a) and (c), the streamfunction obtained by QA, as well as that obtained by SA, shows good agreement with the true solution in both amplitude and structure. Furthermore, the difference in cost function values between QA and CG is at most around $10^{-5}$ after 30 epochs (Fig 8(d)). Even with SA, the cost function (Fig 8(b)) is further improved compared to the case where $n_{x}=10$. These results indicate that when the number of spins is small, the spectral method exhibits favorable performance.

### 3.4. Iteration procedure versus large spin number

So far, we have used “zooming extension” to obtain approximations to real numbers. In this section, we compare this method with the one using a series of spins to express each real number (section 2.5) without iteration. We discretize the domain into a 5 × 5 grid and set ∊ = 0.25 for the Stommel model with finite difference. As an example, we test a case with $n_{spin}=5$ and another with $n_{spin}=8$.

**Fig 9:**
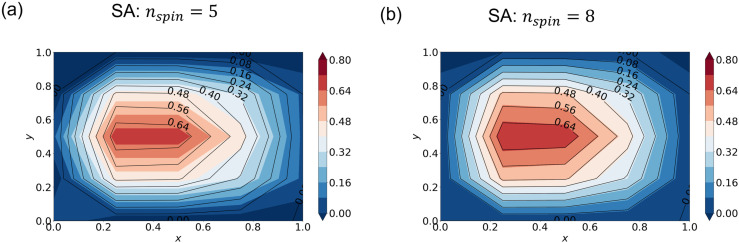
SA solutions using a series of spins without iteration. Streamfunction from SA (shading) and the “true” solution obtained by the standard linear algebra (black contour lines). The hyperparameters are (a) $n_{spin}=5$ and (b) $n_{spin}=8$.

As expected, the SA solution approaches the true solution obtained with standard linear algebra as $n_{spin}$ is increased. The $n_{spin}=5$ solution is significantly different from the true solution (Fig 9a) while the $n_{spin}=8$ solution is very close (Fig 9b). The cost functions for each case are order of $10^{-1}$ and $10^{-2}$, respectively. In contrast, we needed 10 iterations (epochs) with $n_{spin}\;=\;2$, to reduce the cost function to the same level as $n_{spin}=8$ without iteration, indicating that increasing the number of spins is more efficient than the zooming extension.

**Fig 10:**
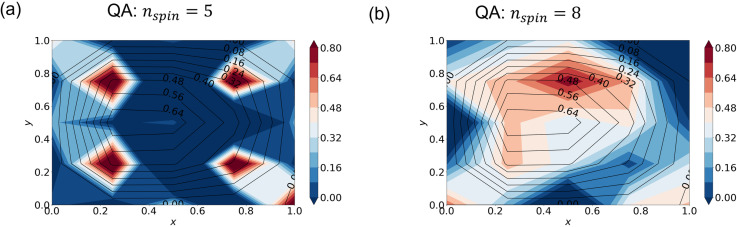
QA solutions using a series of spins without iteration. Streamfunction from QA (shading) and the “true” solution obtained by the standard linear algebra (black contour lines). The hyperparameters are (a) $n_{spin}=5$ and (b) $n_{spin}=8$.

By contrast, the QA method does not produce a good solution without iteration (Fig 10) even though the annealing time is increased to 1000 μs from our standard 20 μs. The cost function remains on the order of 1.0 in this case. The failure can be attributed to noise as well as to the graph embedding problem or discretization error (see also Section 3.2). According to [19], the D-Wave annealer possibly performs worse than the classical computer when the annealing time is on the order of 100μs or longer. A larger number of spins generally requires more annealing time, but noise on the hardware reduces the likelihood of attaining the global minimum.

## 4. Summary

In this study, we have explored potential feasibility and potential problems of the annealing approach for atmospheric and oceanic problems. We consider linear equations, L[f] = 0, and obtain (approximate) solutions to them that minimize the cost function H = ‖L[f]‖. We discretize the cost function in two ways. The first is standard finite-difference and the other is truncated spectral expansion. After the discretization, the cost function takes the form of a quadratic form (Equations (6) and (13)). The solution we seek is a set of real numbers $\left\{w_{1},\;w_{2},\;.\;.\;.\;\right\}$. Quantum annealing, however, operates on spin variables $\left\{\sigma_{1},\;\sigma_{2},\;.\;.\;.\;\right\}$, which take values of either 1 or −1. There are two methods to solve for *w*’s using *σ*’s. One is to approximate each $w_{i}$ as a binary expansion using σ’s (Equation (17)). The other is to search for the solution $w_{i}\;$by iteration named “zooming extension” using a smaller number of $\sigma_{i}$ for each $w_{i}$ at each iteration (section 2.5). That is, the latter method uses fewer spins at the cost of iterations. Here lies a trade-off point: the time it takes for an annealing machine to reach a good solution grows rapidly with the number of spin variables the problem uses. Using either method, the cost function is rewritten into a quadratic form in terms of spin variables. This form is often called “Ising Hamiltonian”. As a quadratic form, an Ising Hamiltonian is written as $H=\sum_{i,j}J_{ij}\sigma_{i}\sigma_{j}+\sum_{i}h_{i}\sigma_{i}$. Matrix entry $J_{ij}\;$represents connectivity between spins *i* and *j*; and where $J_{ij}=\;0$, there is no connection between those spins. As such, the non-zero parts of *J* can be thought of forming a connectivity “graph”. Vector h represents the external field on each spin.

We compared simulated annealing (SA) and quantum annealing (QA), which solve the minimization problem of an Ising Hamiltonian using a simulated and idealized physical process. Each method includes an adjustable parameter that must be increased to obtain a good solution as the number of variables increases, which slows down the simulation. Apart from the fact that QA is carried out on a real machine whereas SA is a software simulation, the most significant difference between QA and SA to our problems is that real machines can handle only very limited connectivity that is hardwired to them. For this reason, we often have to transform the actual graph J of the problem into a graph that can be embedded in the machine’s native graph (Fig 1), which introduces extra spin variables and increases the potential for incorrect solutions (Section 2.6).

We applied QA and SA to the Stommel problem as an illustrative example of simple oceanographic problems. With the finite differential method, the SA approach gave accurate solutions (Figs 3–4). We furthermore demonstrated that appropriate hyperparameters enhance the accuracy more efficiently than merely increasing the number of spins. QA, on the other hand, gave incorrect solutions when the special resolution of finite differencing was high (corresponding to $M^{\prime }\sim 10^{2}$), while QA gave a relatively accurate solution when the resolution was low (corresponding to $M^{\prime }\sim 10$) (Fig 5 and Fig 6). (Of course, the solution is very crude because of the extremely low spatial resolution.)

In the case of spectral expansion as well, QA exhibited poorer performance than SA. When the number of basis functions was 20, QA was able to reproduce the gyre structure; however, the amplitude was smaller compared to the true solution, and the cost function converged to an incorrect value. When the number of basis functions was reduced, QA and SA approximated the true solution with nearly the same level of accuracy. These observations further indicate that the number of qubits has a significant impact on the performance of QA.

Furthermore, we compared the iteration method with the non-iterative method that uses a larger number of spins. QA failed to reproduce the solution even for a coarse problem without iteration (Fig 10). The larger number of spins must have been the ultimate cause of the failure. The larger number of spins requires more annealing time (section 2.4); in addition, it further complicates the graph embedding, further increasing the number of spins (section 2.6). However, SA reproduced the true solution by the non-iterative method (Fig 9), suggesting that future improvements in QA hardware will make QA more practical. If thermal noise and other imperfections are suppressed and better embedding algorithms are developed or a native graph that is closer to that of the problem becomes available, QA will be potentially faster than SA for some problems [6,18–20].

Although we represent real numbers by the iteration procedure following [26], other representations should be evaluated in future work. For example, a recent study [48] proposed a new method to represent real numbers for annealing. The implementation of the new method on our minimization problem and comparison with the zooming extension are interesting future work.

In this paper, we have focused on QA. The other major approach, quantum-gate computing, also has the potential to solve oceanic and atmospheric problems [9,24]. Recent studies have successfully solved nonlinear fluid-dynamical problems based on quantum-gate algorithms [49–51]. Tensor networks can also be used to solve nonlinear fluid-dynamical problems [52–54]. Although the tensor network is a classical algorithm, it can be implemented on quantum-gate machines [52–54]. There are still many challenges before weather and climate simulations become feasible on quantum computers [9]. It is an exciting time.

## Supporting information

S1 Text Appendix 1. Extension of truncated spectral expansion to nonlinear differential equations.
(DOCX)

S2 Text Appendix 2. An intuitive introduction to quantum annealing.
(DOCX)

S3 Text Appendix 3. Uniqueness of the Stommel solution.
(DOCX)
