# Supplementary material for: Formulation and evaluation of ocean dynamics problems as optimization problems for quantum annealing machines
Source: PLoS One. 2025 Jun 26;20(6):e0326303. doi: 10.1371/journal.pone.0326303 (PMC12200861; doi:10.1371/journal.pone.0326303)
Supplement: S1 Text — Extension of truncated spectral expansion to nonlinear differential equations. (DOCX) [file pone.0326303.s001.docx]

***Supporting Information***

*for the paper*

**Formulation and evaluation of ocean dynamics problems as optimization problems for quantum annealing machines**

Takuro Matsuta^a^ and Ryo Furue^b^

^a^ Faculty of Environmental Earth Science, Hokkaido University, Hokkaido, Japan.

^b^ JAMSTEC, Yokohama, Japan.

*Corresponding author*: Takuro Matsuta ([matsuta@ees.hokudai.ac.jp)](mailto:matsuta@ees.hokudai.ac.jp))

Supporting Information: Appendix 1. Extension of truncated spectral expansion to nonlinear differential equations

This appendix is an aside and can be safely skipped; our conclusions are not affected. As we have seen in Section 2, a linear equation can be transformed into a minimization problem of a quadratic form like Equation $\left( 6 \right)$ and renders itself amenable to annealing. What about nonlinear problems as more realistic ocean and atmospheric problems are? At this moment, we cannot handle realistic nonlinear problems; instead, we use an abstract simple nonlinear differential equation to illustrate how the truncated spectral expansion approach can be applied to nonlinear differential equations. We consider a simple nonlinear ordinary differential equation defined in $x\in[0,1]$

$$\begin{aligned} \left( \frac{dy}{dx} \right)^{2}-4x^{2}=0,\#\left( S1 \right) \end{aligned}$$

with the boundary conditions that $y\left( 0 \right)=1$ and $y\left( 1 \right)=2$. The true solution of this equation is $y_{\mathrm{true}}=1+x^{2}$. We expand the solution in terms of basis (as in Equation $\left( 9 \right)$). Here, we employ polynomials $\phi_{m}=x^{m}$ as the basis and expand the solution as

$$\begin{aligned} y=\sum_{m=0}^{n_{basis}-1} w_{m}\phi_{m}.\#\left( S2 \right) \end{aligned}$$

We use Equation $\left( 17 \right)$ to express $w_{m}$ in terms of binary variables. As before (section 2.2.2), we seek an approximate solution that minimizes $H = \left\| \left( dy/dx \right)^{2}-4x^{2} \right\|^{2}$. After a straightforward calculation, we find

$$\begin{aligned} H=\sum_{i,j,p,q} {\tilde{J}_{ijpq}w}_{i}w_{j}w_{p}w_{q}+\sum_{i,j} J_{ij}w_{i}w_{j}+\int_{0}^{1} 16x^{4}dx, \#\left( S3 \right) \end{aligned}$$

where

$$\begin{aligned} \tilde{J}_{ijpq}=\int_{0}^{1} \phi_{i}^{'}\phi_{j}^{'}\phi_{p}^{'}\phi_{q}^{'}dx, \#\left( S4 \right) \end{aligned}$$

and

$$\begin{aligned} J_{ij}=\int_{0}^{1} (-8x^{2})\phi_{i}^{'}\phi_{j}^{'}dx.\#\left( S5 \right) \end{aligned}$$

represent interactions. Here, $\phi_{i}^{'}$ indicates the derivative of $\phi_{i}$. The first term of the cost function involves interactions of four spins, which we replace by quadratic polynomials using Ishikawa’s algorithm [1]. This degree reduction algorithm is implemented in *Fixstars Amplify SDK.*

The minimization problem of the cost function $\left( S3 \right)$ is solved using both the SA and QA approaches. We set $n_{basis}=4$, and $(S,n_{spin})=(0.8, 3)$. Note that the exact solution is $(w_{0}, w_{1}, w_{2}, w_{4}) = (1, 0, 1, 0)$ because $y_{true} = 1 + x^{2}$. **Table 1** shows that both SA and QA reproduce the true solution, but the accuracy of QA is lower. The value of the cost function is below ${10}^{-4}$ in QA and ${10}^{-7}$ in SA after 30 iterations.

**Table 1. SA and QA solutions for the simple nonlinear differential equation.** Each Value indicates the expansion coefficient obtained from the SA and QA approaches.

|  | $w_{0}$ | $w_{1}$ | $w_{2}$ | $w_{3}$ |
| --- | --- | --- | --- | --- |
| SA | 1.00 | $-4.04\times{10}^{-4}$ | 1.00 | $-4.01\times{10}^{-4}$ |
| QA | 1.00 | $2.10\times{10}^{-2}$ | 0.964 | $1.85\times{10}^{-2}$ |

Reference

1. Ishikawa H. Transformation of General Binary MRF Minimization to the First-Order Case. IEEE Trans Pattern Anal Mach Intell. 2011;33: 1234–1249. doi:10.1109/TPAMI.2010.91
