# Supplementary material for: Formulation and evaluation of ocean dynamics problems as optimization problems for quantum annealing machines
Source: PLoS One. 2025 Jun 26;20(6):e0326303. doi: 10.1371/journal.pone.0326303 (PMC12200861; doi:10.1371/journal.pone.0326303)
Supplement: S2 Text — An intuitive introduction to quantum annealing. (DOCX) [file pone.0326303.s002.docx]

***Supporting Information***

*for the paper*

**Formulation and evaluation of ocean dynamics problems as optimization problems for quantum annealing machines**

Takuro Matsuta^a^ and Ryo Furue^b^

^a^ Faculty of Environmental Earth Science, Hokkaido University, Hokkaido, Japan.

^b^ JAMSTEC, Yokohama, Japan.

*Corresponding author*: Takuro Matsuta ([matsuta@ees.hokudai.ac.jp)](mailto:matsuta@ees.hokudai.ac.jp))

Supporting Information: Appendix 2. An intuitive introduction to quantum annealing

We provide an intuitive introduction to quantum annealing (QA) for oceanographers and meteorologists who are not familiar with quantum mechanics or statistical physics. We consider the minimization problem of

$$\begin{aligned} H_{0}=\sigma_{1}\sigma_{2}+\sigma_{1}-\sigma_{2}, \#\left( S6 \right) \end{aligned}$$

where $\sigma_{1}$ and $\sigma_{2}$ are “spin variables” that take +1 (upspin) or −1 (downspin). The first term originates from an interaction between qubits 1 and 2. The second and third terms are an external field acting on qubits 1 and 2, respectively. In terms of the notation of the Ising Hamiltonian (Equation (16) in the main text), $J_{12}=J_{21}=-1/2$, $J_{11} = J_{22} = 0$, $h_{1}=-1$, and $h_{2}=1$ in this case. The minimum value of this Hamiltonian is −3, which occurs when $(\sigma_{1}, \sigma_{2}) = (-1, 1)$. In general, the state $(\sigma_{1}, \sigma_{2}, . . . )$ in which the Hamiltonian takes its global minimum is called the "ground state". **Figure S1** is a schematic representation of this Ising model.

To find the ground state by the QA procedure, we first add quantum fluctuation by “transverse field”. This effect is represented by $B\left( s \right)\hat{H}_{i}$ in Equation (21) in the main text. The time-dependent constant $B(s)$ corresponds to the strength of the quantum fluctuation [1]. Initially, the quantum fluctuation is strong, and hence the ground state $\psi$ of the modified Hamiltonian $A(s)\hat{H}_{0}+B\left( s \right)\hat{H}_{i}$ is represented as the superposition of all possible spin configurations,

$$\begin{aligned} \psi(t=0)=\frac{1}{2}\left( \psi_{\uparrow\uparrow}+\psi_{\uparrow\downarrow}+\psi_{\downarrow\uparrow}+\psi_{\downarrow\downarrow} \right). \#\left( S7 \right) \end{aligned}$$

Here $\psi_{\uparrow\downarrow}$, for example, indicates the spin configuration where qubit 1 is upward and qubit 2 is downward. Equation $\left( S7 \right)$ indicates that each possible spin configuration is observed with a probability of $1/4$ initially (**Figure S2**). We then decrease $B(s)$ to zero and increase $A\left( s \right)$to one sufficiently slowly. As a result, the quantum superposition diminishes and converges to the ground state, $\psi_{\downarrow\uparrow}$, (**Figure S2**), of the original Hamiltonian, $H_{0}$. The adiabatic theorem [2] guarantees the convergence to the ground state of $H_{0}$ under the condition of Equation (22) in the main text.

In summary, QA searches for the global minimum of the cost function using the quantum superposition of $2^{N}$ possible spin configurations simultaneously, where *N* is the number of qubits. The worst-case time QA takes is $\sim\exp\left( N\left| \log\epsilon\right| \right)$ according to Equation (23) in the main text, but if QA takes only a polynomial time, that is, $O(N^{p})$, QA will be faster for sufficiently large *N* than on classical machines, which requires $\mathcal{O(}2^{N})$ calculations in general. Even in the worst cases, QA may still be faster if ϵ is sufficiently small.


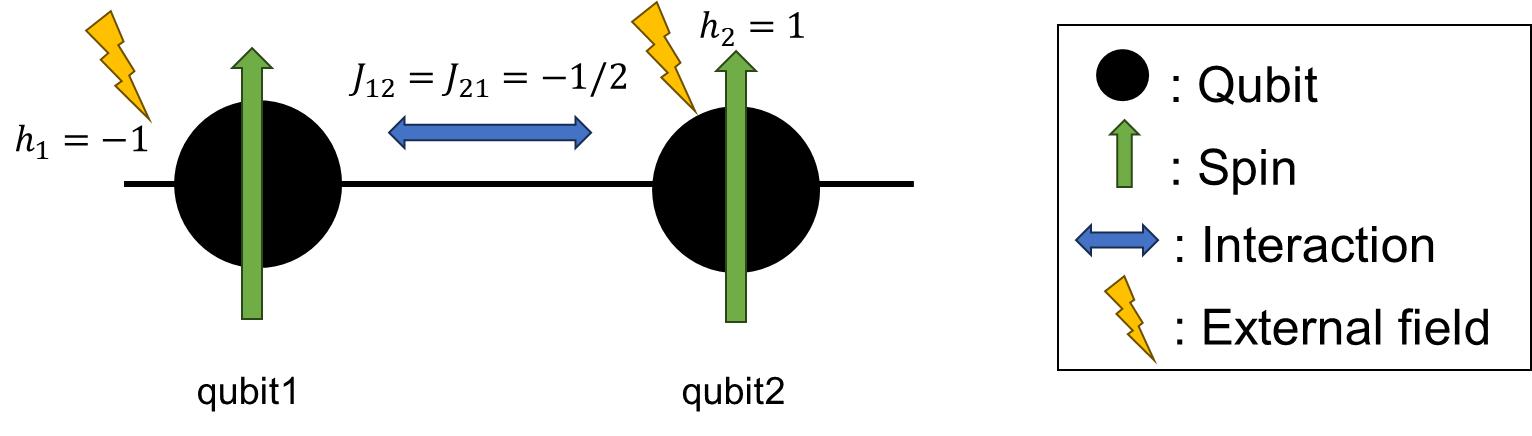


**Figure S1.** **Schematic of the Ising model associated with Equation** $\left( S1 \right)$**.** The figure shows the Ising model when both spins are upward. Green arrows indicate the spins.


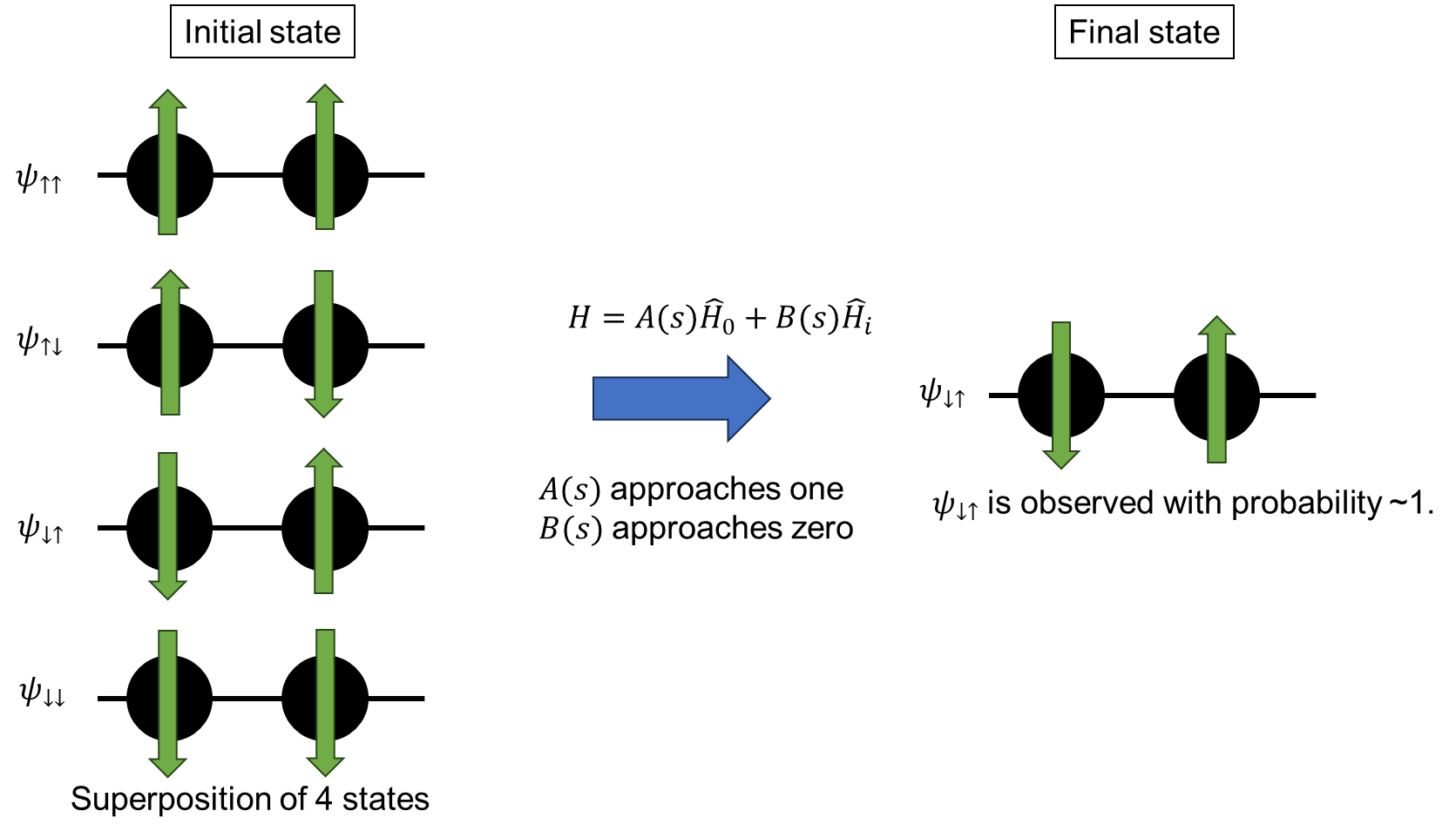


**Figure S2.** **Schematic of the QA procedure for the Ising model of Figure S1.** Left panel indicates the initial state of the QA and the right panel indicates the state after the QA.

References

1. Tanaka S, Tamura R, Chakrabarti BK. Quantum Spin Glasses, Annealing and Computation. Cambridge University Press; 2017.

2. Sakurai JJ, Napolitano J. Modern Quantum Mechanics. Cambridge University Press; 2020. doi:10.1017/9781108587280
