# Supplementary material for: Formulation and evaluation of ocean dynamics problems as optimization problems for quantum annealing machines
Source: PLoS One. 2025 Jun 26;20(6):e0326303. doi: 10.1371/journal.pone.0326303 (PMC12200861; doi:10.1371/journal.pone.0326303)
Supplement: S3 Text — Uniqueness of the Stommel solution. (DOCX) [file pone.0326303.s003.docx]

***Supporting Information***

*for the paper*

**Formulation and evaluation of ocean dynamics problems as optimization problems for quantum annealing machines**

Takuro Matsuta^a^ and Ryo Furue^b^

^a^ Faculty of Environmental Earth Science, Hokkaido University, Hokkaido, Japan.

^b^ JAMSTEC, Yokohama, Japan.

*Corresponding author*: Takuro Matsuta ([matsuta@ees.hokudai.ac.jp)](mailto:matsuta@ees.hokudai.ac.jp))

Supporting Information: Appendix 3. Uniqueness of the Stommel solution

We show the uniqueness of the solution to the Dirichlet problem for the Stommel equation using the Hopf maximum principle in this section. Assume there are two solutions, $\psi_{1}$ and $\psi_{2}$, to the problem. Define a new function $\phi=\psi_{1}-\psi_{2}$. This function satisfies

$$\begin{aligned} \frac{\partial\phi}{\partial x}+\epsilon\left( \frac{\partial^{2}\phi}{\partial x^{2}}+\frac{\partial^{2}\phi}{\partial y^{2}} \right)=0, \#\left( S8 \right) \end{aligned}$$

and $\phi=0$ at the boundary. The uniqueness of the Stommel solution follows if we can show that $\phi$ is the zero function.

The Hopf maximum principle (e.g., [1]) states that if the function $u=u(x)$, $x=(x_{1},x_{2},\ldots,x_{n})\in\mathbb{R}^{n}$, is of class $C^{2}$ and satisfies the inequality

$$\begin{aligned} \sum_{i,j} a_{ij}\left( x \right)\frac{\partial^{2}u}{\partial x_{i}\partial x_{j}}+\sum_{i} b_{i}\left( x \right)\frac{\partial u}{\partial x_{i}}\geq0, \#\left( S9 \right) \end{aligned}$$

in a domain $\Omega$, where the symmetric matrix $a_{ij}$ is locally uniform positive definite and the coefficients $a_{ij}$ and $b_{i}$ are locally bounded, then $u$ is consntant in $\Omega$ if $u$ takes a maximum value in $\Omega.$

Since $\phi$ is the continuous function defined in the closed domain $\left[ 0,1 \right]\times[0,1]$, $\phi$ should have the maximum and minimum values. We will prove $\phi\equiv0$ by considering the cases where the maximum or minimum of $\phi$ occurs in the interior of the domain and on its boundary. If $\phi$ takes its maximum or minimum in $\left( 0,1 \right)\times(0,1)$, then $\phi$ is constant from the Hopf maximum principle. Since $\phi$ is continuous, $\phi\equiv0$ in $\left[ 0,1 \right]\times[0,1]$ to satisfy the boundary condition. If $\phi$ takes its maximum and minimum at the boundary, both the maximum and minimum is zero due to the boundary condition, resulting in $\phi\equiv0$ in $\left[ 0,1 \right]\times[0,1]$.

Reference

1. Pucci P, Serrin J. The strong maximum principle revisited. Journal of Differential Equations. Academic Press Inc.; 2004. pp. 1–66. doi:10.1016/j.jde.2003.05.001
